# Understanding the Snake Venom Metalloproteinases: An Interview with Jay Fox and José María Gutiérrez

**DOI:** 10.3390/toxins9010033

**Published:** 2017-01-16

**Authors:** Jay W. Fox, José María Gutiérrez

**Affiliations:** 1 Department of Microbiology, Immunology and Cancer Biology, University of Virginia School of Medicine, P.O. Box 800734, Charlottesville, VA 22908, USA; jwf8x@virginia.edu; 2 Instituto Clodomiro Picado, Facultad de Microbiología Universidad de Costa Rica, San José 11501-2060, Costa Rica; jose.gutierrez@ucr.ac.cr

## Abstract

Jay W. Fox and José María Gutiérrez recently finished editing a Special Issue on the topic “Snake Venom Metalloproteinases” in *Toxins*. The Special Issue covers a wide range of topics, including the molecular evolution and structure of snake venom metalloproteinases (SVMPs), the mechanisms involved in the generation of diversity of SVMPs, the mechanism of action of SVMPs, and their role in the pathophysiology of envenomings, with implications for improving the therapy of envenomings. In this interview, we discussed with Jay W. Fox and José María Gutiérrez their research on the SVMPs and their perspectives on the future trends and challenges for studying snake venoms.

Jay Fox is a Professor of Microbiology, Immunology, and Cancer Biology, at the University of Virginia School of Medicine and an Associate Director of the UVA Cancer Center. Dr. Fox currently is engaged in research on carcinogenesis in women with dense breasts focusing on the interaction of stroma and breast epithelium. He is also interested in the secondary or indirect effects of viper envenomation focusing on the roles of venom and host generated damage-associated molecular pattern molecules (DAMPs) in the pathophysiology of snake bites. Dr. Fox directs the Office of Research Core Administration and oversees the operation of 15-shared resource core facilities employing approximately 60 faculty and staff. Dr. Fox teaches courses to both medical and graduate students on cancer biology and also teaches a course on research ethics. He has served as the President of the Association of Biomolecular Resource Facilities, was a member of the Federation of Associations of Experimental Biology, and is currently serving as the President of the International Society on Toxinology. Dr. Fox participated on numerous NIH Study Panels and sits on the External Advisory Committees for two National Cancer Institute Designated Cancer Centers. Outside of work, he enjoys being a Scoutmaster for Troop 37 in Charlottesville, Virginia and sailing and oyster ranching at his home on the Chesapeake Bay (Figure 1).

José María Gutiérrez is a Professor at the University of Costa Rica, where he performs research on snake venoms and antivenoms at the Instituto Clodomiro Picado and teaches Immunology, Research Methods, Cellular Pathology, and Biochemistry at the School of Microbiology. Dr. Gutiérrez’s main research interests are related to the composition and mechanism of action of snake venom toxins, particularly regarding metalloproteinases and phospholipases A_2_ responsible for the drastic local tissue damage characteristic of viperid snakebite envenomings. Dr. Gutiérrez is also involved in the development of novel antivenoms for various regions of the world and in the preclinical evaluation of antivenom efficacy, as well as in the search for novel inhibitory compounds that could be used to treat envenomings. Dr. Gutiérrez is interested in public health aspects of snakebite envenoming as well, and participates in extension programs aimed at improving the prevention and management of snakebites in Costa Rica and abroad. For his contributions, Dr. Gutiérrez has received several national and international awards, including the Redi Award (2015) of the International Society on Toxinology (Figure 1).

**Q. When did you first become interested in snake venoms and how did you get involved in research on this topic?**

**Jay Fox:** I began my academic life as an undergraduate student at Monmouth College studying biology, chemistry, and philosophy. While I had no idea at the time what career I wanted to pursue, I knew I loved science. As I neared the end of college I took a deep interest in organic and biochemistry and my advisor Dr. John Kettering suggested I consider graduate school. None of my family had progressed beyond a bachelor’s degree so I had no idea what this entailed but since I had no other plans…why not? I was accepted at Colorado State University and matriculated without concern mainly based on total ignorance of just what earning a Ph.D. would entail or for that matter what it would ultimately prepare me for in terms of a career. As a first year graduate student studying biochemistry at Colorado State University, a more senior student, Jon Bjarnason, who was in Professor Anthony Tu’s laboratory, befriended me. Jon was studying snake venoms and told me how interesting it was trying to isolate toxins and understand their mechanism of action. Dr. Tu’s laboratory was very well equipped and I met with him and we discussed possible projects. One was to isolate sea snake neurotoxins and in order to collect the venom I needed, he would send me to Asia. That sounded very exciting given that I had never travelled much so I signed on with Dr. Tu. As it turned out it was an excellent decision in that I not only learned about venoms and toxins, but I also received an excellent education on protein chemistry and protein structure and function which has served me well throughout my career regardless of what biomedical subject I am investigating.

After graduating I did post-doctoral work first in the laboratory of Dr. Marshall Elzinga at Brookhaven National Laboratory, Upton, N.Y. and then with Professor John Stewart at the University of Colorado Medical School, Denver, Colorado. Dr. Elzinga was a superb protein chemist who was a leader in sequencing large muscle proteins. He had just moved to Brookhaven when I arrived and together we set up his spinning cup Edman sequencer. To identify the amino acids from the sequence we did a combination of thin layer chromatography and back hydrolysis of the phenylthiohydantoin (PTH)-amino acids using a home built amino acid analyzer Dr. Elzinga acquired from Dr. Stein at the Rockefeller University. While I was there we also began using the new technique of HPLC to analyze the PTH amino acids. Brookhaven at the time was a focal point of outstanding protein studies and it was a wonderful experience and I met many leaders in the field of protein characterization. At Colorado, working with Professor Stewart was also an honor and privilege for me. John had previously worked with Nobel Prize winner, Professor Bruce Merrifield, developing an automated peptide synthesizer. John knew all about peptide synthesis, synthesizers and peptide isolation. John’s book on the subject was in every synthesis lab at the time and always well-worn with use. My project as an NIH fellow in John’s lab was to synthesize novel ACTH analogs looking for novel activities. John was a very generous scientist giving me time to work on my own ideas as well. One project I conducted was to synthesize the active site loop of a sea snake neurotoxin to determine if the peptide could recapitulate some of the neurotoxin’s activities. The project was successful as we made an active peptide, but I never published it as my formal project with John took precedent with my time. Hence, as I say later in this piece, if you do not publish your work it did not happen…at least as far as the rest of the world is concerned. 

While finishing up my studies in Denver I had two job offers, one at the University of Virginia and one at the Coors Brewery in Golden, Colorado. I was tormented with this choice; to go into academics or stay in Colorado, a place I loved for its hiking and backpacking. I chose Coors, but on the day I was to show for work, I had a change of heart and called Virginia and told them I would be there in two weeks. Over the first few years I often wondered if I had made the correct decision. Now nearly four decades later, it was clearly the best decision of my life.

I arrived at the University of Virginia School of Medicine as an Assistant Professor of Microbiology with the charge to bring modern protein chemistry technology to the school. This I did by starting a protein-sequencing core, then a peptide synthesis core, and eventually a DNA sequencing core. These activities solidified in my mind the value of utilizing cutting edge technology applied to whatever area you may be studying. Simultaneously I pursued my interests in toxinology, beginning with isolating a number of snake venom metalloproteinases, characterizing them and ultimately determining their protein and cDNA sequences, which were some of the first ever published. Over the intervening years I have strived to always do something novel in the field following the admonition of my sabbatical host at the Max-Planck Institute for Biochemistry in Munchen, Dr. Rupert Timpl, who always said with regards to a project “You must do it first or do it a lot better; generally it is easier to do it first”. Something I always tell my students as well when thinking about projects.

**J.M. Gutiérrez:** I started working as an undergraduate research assistant at the Instituto Clodomiro Picado in 1975, under the supervision of Róger Bolaños, the founder and first director of this institute. As a mentor, Dr. Bolaños instilled in me the vision that research can be done with passion and joy, and also the belief in the relevance of the social implications of scientific work, in this case in relation to snakebite envenomings and the human suffering they inflict. This went hand in hand with my own social and political beliefs. My first research projects had to do with the study of karyotypes of venomous snakes, but rapidly I became interested in venoms and antivenoms. At that time relatively little was known on the pathogenesis of the local tissue damage induced by viperid snake venoms, a very important aspect of snakebite envenoming since it may lead to permanent tissue damage and other sequelae in the victims. Initially, I studied the local pathology induced by Costa Rican snake venoms in mice by using light microscopic techniques. In 1980, I had the opportunity to perform my PhD studies under the supervision of Prof. Charlotte L. Ownby at Oklahoma State University, with the support of a scholarship provided by the University of Costa Rica. Charlotte made significant contributions to the study of venom-induced pathology. In her laboratory we were able to isolate and characterize a myotoxic phospholipase A2 from the venom of *Bothrops asper*, the most important snake in Central America. In addition, we studied the action of this toxin in muscle tissue, by using electron microscopy and other techniques, and proposed a mechanism of action for myotoxic phospholipases A2. In 1984 I returned to Costa Rica and continued my research at the Instituto Clodomiro Picado, in collaboration with a highly qualified group of Costa Rican colleagues and international collaborators with whom I have worked for over 40 years. The philosophy of cooperation and partnership that has characterized the work of Instituto Clodomiro Picado, and its relationships with groups in our own country, and in countries of Latin America, North America, Europe, Asia, Africa, and Oceania, has allowed our team to contribute to toxinological research and antivenom development.

**Q. Can you describe your research group’s current work? How has it changed over the past ten years, and where do you see it going in the future? Is there an area of the field that you are particularly excited about at the moment?**

**Jay Fox:** Currently my laboratory focuses on the role of damage-associated molecular pattern molecules (DAMPs) on snake envenomation pathophysiology. We work closely with José’s group in Costa Rica. This area, which we discovered, is going to play an important role in understanding the non-lethal aspects of snake envenomation that are associated with envenomation morbidity. My lab is also working on the role of stroma in carcinogenesis in dense breast tissue. Ironically, there are some features of these areas of research which intersect, such as the role of stroma in envenomation and in carcinogenesis and tumor invasion. While there is certainly a lot yet to discover regarding toxins and their activities, one must admit that much has been learned over the recent past by virtue of the explosion of proteomic, transcriptomic, and now genomic studies on snake venoms and snakes themselves. As we have written, in the end it is ultimately a systems biology issue in terms of how all the toxins in the venom collectively give rise to the effects observed in the host as well as how the biology of the snake and its environment also impinges on what effects the venom may cause. So, for me, the future will be in discovering what previously unknown activities some toxins may have and how all the toxins work together under the biological systems of the snake to give rise to the observed pathophysiology in envenomated hosts. 

**J.M. Gutiérrez:** I participate with several research groups at the Instituto Clodomiro Picado, since we have a cooperative and integrative philosophy of doing research. Specifically on the subject of snake venom metalloproteinases (SVMPs), I work with Alexandra Rucavado, Teresa Escalante, Erika Camacho, and Cristina Herrera, in addition to several graduate and undergraduate students. For many years, we have also collaborated with a number of research groups from other countries; in this particular subject of SVMPs we have had fruitful collaborations with the groups of Jay W. Fox (University of Virginia, USA), Michael Ovadia (University of Tel Aviv, Israel), Catarina F.P. Teixeira and Ana M. Moura-da-Silva (Instituto Butantan, Brazil), and Juan J. Calvete (Instituto de Biomedicina de Valencia, Spain), among other groups. Since the early 1990s our main goal on the topic of SVMPs has been to understand how these toxins induce hemorrhage, one of the main manifestations of viperid snakebite envenomings. Initially we isolated and characterized a number of SVMPs, and studied their action using transmission electron microscopy and other microscopic approaches. Then, we investigated the action of hemorrhagic and non-hemorrhagic SVMPs on the basement membrane of capillary blood vessels, by combining histology, ultrastructure, immunohistochemistry, and immunoblotting. More recently, and in a close collaboration with J. W. Fox, we have introduced the proteomics analysis of exudates collected in the vicinity of SVMP-damaged tissue as a tool to have a deeper view of the pathological alterations occurring in the tissue. As an outcome of these investigations, a model for the mechanism of action of hemorrhagic SVMPs has been proposed, based on the cleavage of structurally-relevant basement membrane components, especially type IV collagen, followed by the mechanical disruption of vessels due to hemodynamic biophysical forces operating in the circulation. In the near future we are interested in the identification of the regions in the molecular structure of SVMPs that determine their ability to bind to microvessels, as well as of the cleavage sites of basement membrane proteins that determine the disruption of capillary blood vessels. Moreover, our more recent studies in collaboration with Jay Fox indicate that fragments of extracellular matrix proteins and other types of proteins released in the tissues as a consequence of SVMP action may contribute to tissue alterations and may play roles in the processes of repair and regeneration, a hitherto unknown subject which may bring novel clues for understanding the pathogenesis of venom-induced tissue damage. An additional challenge for our future studies is to understand the role of SVMPs in envenomings from an integrative perspective, i.e., in the light of the overall picture of envenoming, which involves studying the synergistic actions of SVMPs and other venom components, a poorly studied aspect of envenomings.

**Q. It is well-known that snake venom metalloproteinases (SVMPs) are the primary factors responsible for hemorrhage. How does an improved understanding of actions of the SVMPs advance our understanding of snakebite envenoming? Do you think sufficient research has been done for the SVMPs? Are there any aspects that need further exploration?**

**Jay Fox:** When Dr. Solange Serrano and I coined the name and classifications of the SVMPs, it an incredibly exciting time in the field. For a long period when only limited sequence data was known for the SVMPs (hemorrhagic and non-hemorrhagic alike), Dr. Jon Bjarnason, my close colleague, and I were leaning toward SVMPs being members of the matrix metalloproteinase family and we were pushing for recognition of this classification. However, Dr. Hideaki Nagase told us, based on his studies, these SVMPs were not matrix metalloproteinases (MMPs), and as it turns out, he was right! Also at this time I had the good fortune to hear a talk by Dr. Judith White on a new class of fertility related proteins found on sperm that seemed to have proteinase activity. When she shared some preliminary sequence data on these proteins with me, I had an epiphany; they had sequences similar to the SVMPs! We were no longer alone; there were normal orthologs to these toxins, something which is now very well known about all venom proteins. The class of proteins Judy was working on became known as A disintegrin like and metalloproteinase proteins (ADAMs) and the study of ADAMs and SVMPs exploded hand in hand. SVMPs were often referred to for insights into the structure and function of ADAMs and this is now a paradigm of toxinology in that the study of toxins often provides outstanding insights into the function of their normal orthologs and comparison of the toxins with the orthologs can also yield insights into how small changes in structure and/or location can cause a somewhat “normal” protein to become toxic.

There are still some lingering aspects of SVMPs that deserve study. Certainly their evolution and gene structure merit additional investigation. Also, higher resolution understanding of substrate specificity as associated with functional activity is still of some interest, and as mentioned above, how these data may inform on the ADAMs’ functions is also of value.

**J.M. Gutiérrez:** Many years ago, through the pioneering work of A. Ohsaka, A.T. Tu, J.B. Bjarnason, J.W. Fox, and F.R. Mandelbaum, among other researchers, it was demonstrated that SVMPs were responsible for the hemorrhagic activity of snake venoms. Local and systemic hemorrhages are key aspects of viperid snakebite envenomings, since they are associated with local tissue damage and with systemic bleeding leading to cerebrovascular accident and hemodynamic perturbations. In addition, SVMPs also participate in the alterations of blood coagulation in these envenomings, and release pharmacologically-active components from proteins of the extracellular matrix, which are likely to contribute to tissue damage and repair. Thus, SVMPs are at the center of the pathophysiology of viperid snakebite envenomings. The study of the mechanisms of action of SVMPs has provided valuable clues to our understanding of the overall pathophysiology of these envenomings. Even though there has been a great deal of research on SVMPs, there are still pending issues for understanding their molecular evolution, regulation of expression, mechanisms for generating the great diversity of these enzymes in venoms, mechanisms of action, structural determinants of toxicity, and their overall role in the pathophysiology of envenomings, as well as their ecological role, i.e., their action in natural prey. Likewise, the search for novel and more potent SVMP inhibitors is an area of research that might offer novel therapeutic alternatives. These aspects require renewed investigation in the near future, hopefully through interdisciplinary approaches that will pave the way for more integrative and holistic perspectives of this fascinating group of venom components. 

**Q. Looking back at research on snake venoms, what do you think have been the most important milestones? What are the most important techniques that will contribute to research on snake venoms in the future?**

**Jay Fox:** The key discoveries that have led us to our current understanding, in my opinion, are in this order:

(a) Venoms are comprised of toxic and non-toxic components (many of which are proteins and peptides).
(b) In many venoms, the activity of toxins is dependent on enzymatic activities directed at specific substrates.
(c) In the case of the SVMPs, the hemorrhagic SVMPs were demonstrated to disrupt the basement membranes of capillaries allowing the extravasation of contents into the stroma. This finding was critical in that it focused our research on the proteolysis of matrix by SVMPs and how this is related to the observed pathology (a topic we continue to study by our current focus on the roles of DAMPs in snake envenomation).
(d) Venoms are somewhat complex and this is demonstrated by proteomics and transcriptomics.
(e) The toxic effects of venom are due to the collective action of toxins and non-toxic components in the venom and to understand envenomation one must ultimately take a systems approach.
(f) Development of anti-venom agents must always consider the venom as a system; not a simple collection of unilaterally acting toxins.
(g) By discovering the activities of toxins we will better understand the function and structure of normal orthologs, and by understanding the targets and the nature of the toxin mechanisms, the possibility of developing novel drugs becomes a reality.

Certainly, the techniques of omics will continue to be important in venom studies as well as advanced microscopy; however, I would also suggest that modern physiology and molecular physiology will play an increasingly important role if we are to fully understand the function of individual toxins as well as venoms.

**J.M. Gutiérrez:** Over the last decades there have been a number of relevant achievements in our understanding of snake venoms, their molecular evolution and composition, mechanism of action and identification of their targets in tissues and blood, and ecological and medical roles. Likewise, the understanding of the structure-function of many snake toxins has paved the way, as has the study of other animal toxins, to the discovery of promising leads which may derive into new drugs for the treatment of a variety of diseases. It is the combination and integration of molecular evolution and structural studies, in parallel with the characterization of venom proteomes and the understanding of the structure-function relationship, as well as the mechanism of action of many toxins, that I consider major achievements in the field of toxinology.

Many techniques will impact on the future of toxinology. The growing impact of the ‘omics’, with the power of bioinformatics, is generating and will continue to provide vast amounts of information on snake venoms, but this has to be linked to the study of the mechanisms of action of venom toxins from both ecological and medical perspectives. It is the integration of such vast volumes of information, from a Systems Biology perspective, that will bring a more complete understanding of SVMPs and venoms in general from the evolutionary, ecological, and medical perspectives. The ongoing work on the genomes of several snakes is an exciting development, together with the completion of novel venom proteomes and venom gland transcriptomes, the structural characterization of novel venom’s toxins, and the understanding of tissue targets and mechanisms of action, with the aid of many techniques, including recent developments in imaging.

**Q. Are there any challenges for future research on snake venoms?**

**Jay Fox:** There are several challenges to snake venom research facing us. First, the easy work has been done. What is left requires very clever, insightful thinking and a judicious use of resources and application of instrumentation/technology. Simply isolating additional isoforms of venom components is of little impact unless one has very clear and clever ideas about novel activities for these isoforms and ways to test for them. Also, there is the risk that we lose sight of why we are doing this research. We must always remember it is to advance science and as such we should be improving the human condition. Whether we are doing basic, translational, or clinical research we must always be able to connect what we do, in a coherent fashion, to improving the human condition. And, we must be able to explain this to the lay public. If we cannot do that we will just become a group of disaffected hobbyists without impact or interest to the wider world.

**J.M. Gutiérrez:** The main challenge, in my view, is how to integrate a massive volume of data that is being generated on snake venoms in order to provide understanding in addition to information. A big challenge is to develop inter- and transdisciplinary research approaches aimed at generating deeper insights on the evolutionary, ecological, and medical aspects of snake venoms. The field of toxinology should move in the future from a predominantly ‘reductionist’ approach to a more ‘holistic’ perspective of snake venoms. Another big challenge for the future is to reduce the gaps between basic toxinological research and the clinical and antivenom manufacturing areas, i.e., to generate more translational research in toxinology. The cross-talk between basic toxinologists and clinical toxinologists and with antivenom developers is of paramount relevance in order to bring the scientific advances to the improvement of the treatment of envenomed patients. 

**Q. How do you anticipate research on snake venoms will progress over the coming years?**

**Jay Fox:** I believe there will be more drugs developed based on research stemming from venoms by virtue of new target discovery as well as new activities found for some toxins. I also believe we will become much more efficient and effective in developing antivenoms (by virtue of the work of my colleagues in Jose’s group and others) based on a scientific understanding of venoms and venom action. Also, with the closer association of a number of regional societies with interests in toxinology with the International Society on Toxinology, I believe all the science in the arena will be enhanced with an improved outcome for those who suffer from envenomation both in terms of health as well as financial burdens. 

**J.M. Gutiérrez:** I foresee advances in the following areas:

(a) Vast volumes of new information on snake genomes, venom gland transcriptomes, and venom proteomes, all of which will enlighten our comprehension of snake venom evolution.
(b) A deeper understanding on the structure-function of venom components, particularly in those playing a key role in toxicity. Additionally, identification of the key toxic components in medically-relevant venoms, with the consequent impact in the design and evaluation of antivenoms.
(c) Significant progress in our understanding of the mechanisms of action of toxins and identification of their targets, with implications both for clinical management of envenomings and for drug design.
(d) Deeper understanding of the ecological role of venoms, i.e., promotion of stronger links between toxinology and natural history.
(e) Exploring poorly studied venoms, such as those of ‘colubrid’ snakes (*sensu lato*) and other taxa, with the aim of discovering new types of toxins and mechanisms of action. Such novel toxins might become useful tools to understand basic physiological processes and as sources of new lead compounds for drug design.
(f) Harnessing the vast volume of toxinological information for improving the design and development of antivenoms, either animal-derived products or novel antivenoms based on recombinant antibody technology. Additionally, the discovery and development of molecules with strong inhibitory action that could complement the action of antivenoms in the therapy of envenoming.
(g) Developing the field of Public Health within the area of snakebite envenoming in order to understand the role of snake venoms in disease from a broader perspective.

**Q. For the Special Issue “Snake Venom Metalloproteinases” that you edited for *Toxins*, what new information do you think it has brought forward and what gaps in the literature has it filled? How will our current research on SVMPs shed light on the future snakebite envenoming therapy?**

**Jay Fox:** This Special Issue represents an outstanding mix of comprehensive reviews, where many such gaps have been addressed, as well as new research opening novel areas of scientific pursuit in toxinology, such as the work on DAMPs by Rucavado and colleagues and concepts on serine proteinases by Kini and colleagues. I personally would recommend this Special Issue to all new students to the field and to those more senior who wish to understand what is the current state of thinking in the field. 

I am certain a careful reading of this Special Issue will sharpen our understanding of snake envenomation and it is my sincere hope that this will be translated to new science and new modes of therapy for both snake envenomation as well as possible new drug leads for other diseases.

**J.M. Gutiérrez:** This Special Issue includes highly relevant contributions that present the state of the art in SVMP research. The principal aim of this Special Issue is to provide a synthesis of our current knowledge of this fascinating field of toxinology. In addition, novel findings are presented which bring new concepts to the field of SVMPs. The readers will find in these contributions the key issues behind our current knowledge on SVMPs, with highly suggestive views on several unsolved aspects in SVMP research. The papers cover a wide range of topics, from molecular evolution and structure to the mechanisms involved in the generation of SVMP diversity, from the mechanism of action of SVMPs to their role in the pathophysiology of envenomings, with implications for improving the therapy of envenomings. Any person interested in SVMP will find in this Special Issue a collection of papers that summarize the current knowledge on these types of enzymes, and at the same time presenting the most relevant open questions in this topic, as a stimulus for future research efforts.

The understanding of SVMP variability and structure, as well as mechanisms of action and targets, will undoubtedly provide highly useful information for improving the therapy of snakebite envenoming, particularly in the case of species, such as the majority of viperid snakes, whose pathophysiological activities largely depend on the action of SVMPs. Understanding the structural determinants of toxicity, the targets of these toxins in the tissues, and their toxicokinetic profiles will lead to renewed knowledge-based antivenom design, as well as to the development of novel recombinant antivenoms and new inhibitors of high efficacy that could be used as a first aid in the field as a complement to antivenom therapy.

**Q. What advice do you have for a young scientist who wants to start a career in snake venom research?**

**Jay Fox:** Frankly, I must give much of the same advice I was given by my mentor Professor Tu. Some things do not change. Regardless of what you are investigating, snake venoms or whatever, you must always be thinking not only about how your work will impact this field but how it can have impact beyond your field; and ultimately how it can affect the human condition. The critical thinking, the sophisticated tools, experimental design, and data analysis involved in high quality venom research is no different than that required in all fields. It is applicable in all domains. Whatever you may be researching in the field of toxinology, always do so looking over the horizon to see how what you are learning can be applied to other fields and what scientists in other fields are doing that you can co-opt for your use in your studies. Science in a vacuum is irrelevant. You must present your work; you must publish your work. Someone spent their hard-earned money trusting you to do good work for the good of the world. Do not waste their money and do not waste your time. Make it count. Also, collective science working with a variety of colleagues often gives rise to the best science and likely is a lot more enjoyable. Science is too complex now to do it all alone.

Finally, have fun and if should you lose interest in a field, then move to another; and should you lose interest in science, there are many other admirable things to do. Find your passion and follow it.

**J.M. Gutiérrez:** There are many points of advice I would give to young people interested in snake venom research. Some of them are:

(a) Have a broad knowledge and understanding of the field of snake venoms. In addition to specializing in a particular type of toxin or venom, understand snake venoms from a wide perspective, i.e., their biological relevance, evolution, variation, mechanisms of action, and medical significance. Such broad views will help you place your particular questions in a larger landscape and will allow you to generate more relevant questions for your research. For this you need to follow the scientific literature in the subject on a regular basis and with discipline.
(b) Find topics for your research which are, at the same time, exciting for you and novel. Avoid the ‘me too’ type of research which may bring abundant data but few new ideas, and try to find unexplored or poorly explored niches where your contributions will be more meaningful. For this, it is necessary to have an ample knowledge on snake venoms (see item (a) above).
(c) Independently of your specific research interests, cultivate your knowledge in general biological and biomedical topics such as evolutionary biology, biochemistry, bioinformatics, structural biology, cellular biology, and physiology, as well as pathophysiology, among others. Having a background in these general subjects will allow you to place your toxinological research interests in a broad scenario which, in turn, will more likely provide you with new ideas.
(d) As much as possible, try to think ‘out of the box’. This will give you the opportunity to generate new ideas and concepts, and new experimental tools and models. The collection of experimental data should be guided and complemented by the generation of new ideas and hypotheses. When possible, try to get out of the predominant paradigms and take the risk of generating new ways of looking at things, regardless of the natural resistance that this may provoke. After all, scientific work is, to a large extent, about generating novel ways to view the topics that are being investigated.
(e) Develop a cooperative philosophy for doing scientific research. Be part of interdisciplinary teams and groups in order to approach questions of wide interest from your own area of expertise. Be humble and accept that you need the contribution of other scientists for developing your own research projects. Be in contact with colleagues and actively discuss subjects of common interest with them. Procure the people that can help you and, at the same time, be generous with your colleagues and students by sharing your knowledge. Keep in mind that scientific research is a collective undertaking.
(f) Finally, in addition to being a dedicated scientist, strive to become also a person interested in general issues related to society and culture as a whole. Scientists have a huge social responsibility, and toxinologists are no exception. Develop a compassionate and generous way of doing science and in shaping your relation with societal issues in general. Introduce and follow ethical considerations when doing scientific research and in your life in general.

## Figures and Tables

**Figure 1:**
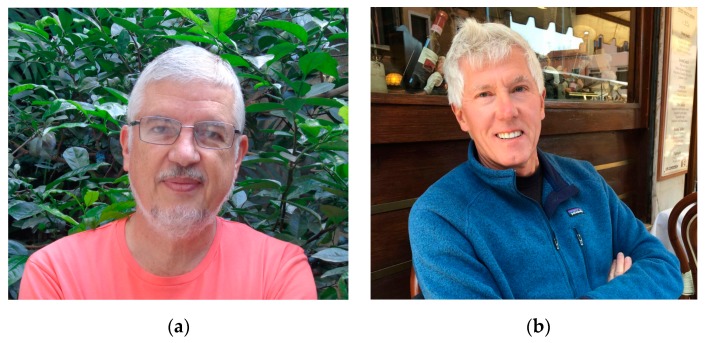
(**a**) José María Gutiérrez; (**b**) Jay W. Fox.

